# 

**Published:** 1832-07-01

**Authors:** 


### MEDICO-CHIRURGICAL

### REVIEW,

AND

### JOURNAL

OF

### PRACTICAL MEDICINE.

*(NEW SERIES.)*

VOLUME SEVENTEEN,

[1st of APRIL to 30th of SEPTEMBER,]

1832.

VOL. XXI. of ANALYTICAL SERIES.

EDITED BY JAMES JOHNSON, M.D.

PHYSICIAN EXTRAORDINARY TO THE KING,

*&c. &c. &c.*

LIBRARY. 57456

LONDON:

PUBLISHED BY S. HIGHLEY, 32, FLEET STREET,

AND WEBB STREET, ST. THOMAS'S HOSPITAL,

And all other Booksellers.

W1
ME 75T

PRINTED BY G. HAYDEN,

Little College Street, Westminster.

### BIBLIOGRAPHICAL RECORD;

OR,

*Works received for Review since the last Quarter.*

1. The Dissector's Guide; or, Student's Companion. Illustrated by numerous Wood-cuts, clearly exhibiting and explaining the Dissection of every part of the Human Body. By EDWARD WILLIAM TUSON, F.L.S. &c. Small 8vo, boards, pp. 219. Wilson, London, 1832

☞ *Mr. Tuson's myological and other anatomical works are already such favourites with students, that we do not doubt his success in the present attempt. We have looked over this Dissector, and we think that the wood-cuts which illustrate the description and explain the directions are likely to prove extremely useful to the young dissector. The price is very moderate—nine shillings. We should mention that the work contains the methods of securing the different arterial trunks, and a brief account of the fractures and dislocations, &c.*

---

2. An Account of Cholera and Cholera Fever, as they existed at Rochester and its Vicinities, in the Year 1818. Published at that time by the late WALTER VAUGHAN, M.D. Licentiate of the College of Physicians of London. Octavo, stitched, pp. 54. 1832.

☞ *We may notice this.*

3. Observations on some diseases of the Vascular System. Essay I. Diseases of the Veins. Essay II. Diseases of Capillaries. By THOMAS A. WISE, M.D. Bengal Medical Service, &c. Octavo, stitched, pp. 96—23. Calcutta, 1831.

☞ *This we shall also take an opportunity of noticing.*

---

4. The Mother's Medical Guide; containing a Description of the Diseases incident to Children; with the Mode of Treatment, as far as can be pursued with safety, independently of a Professional Attendant. By the late ROBERT BRADFORD and H. O. BRADFORD, M.R.C.S. &c. Octavo, stitched, pp. 96. Price 3s. 6d.

---

5. The Principles and Practice of Obstetric Medicine, &c. By DAVID D. DAVIS, M.D. &c. Part VI. Price 2s. One Lithographic Plate.

☞ *This shall be noticed in the Periscope Department of the present number.*

---

6. Hyperanthraxis; or the Cholera of Sunderland. By W. REID CLANNY, M.D. F.R.S.E. &c. Octavo, pp. 149. London, 1832.

7. Two Lectures on the Circulation, Respiration, and Mode of Nutrition in Animals and Plants, &c. Delivered before the Chesterfield Literary and Philosophical Society. By W. H. ROBERTSON, M.D. Octavo, stitched, pp. 32. Price 1s. 6d.

☞ *A popular and interesting view of what is an interesting, and should be a popular subject.*

---

8. A Practical Treatise on Uterine Hæmorrhage in connexion with Pregnancy and Parturition. By JOHN T. INGLEBY, M.R.C.S. Lecturer on Midwifery at the School of Medicine at Birmingham, &c. 8vo, pp. 276. London, 1832.

---

9. On the Nature and Cure of Glandular Diseases, especially those denominated Cancer, with the Mode of Treatment; and on the too frequent use of Mercury, &c. Also, on Organic Affection of the Stomach, with a few Remarks on Cholera, &c. By Sir CHARLES ALDIS, Surgeon and Accoucheur, &c. Octavo, pp. 116. London, 1832.

---

10. Histoire Générale et Particulière des Anomalies de l'Organization chez l'Homme et les Animaux. Ouvrage comprenant des Recherches sur les Caractères, la Classification, l'Influence, Physiologique et Pathologique, les Rapports Généraux, les Lois et le Causes des Monstrosites, des Varietés, et Vices de Conformation, ou Traité de Tératologie. Par M. ISIDORE GEOFFROI SAINT-HILAIRE, Docteur en Médécine, &c. Tome 1er, pp. 746, avec Atlas, in 8vo, xx. Planches. Paris et Londres, chez Baillière. 1832.

☞ *We shall notice this able production.*

---

11. Substance of the Investigation regarding Cholera Asphyxia: with Cases and Dissections, communicated by Professor Delpech, and Dr. Coste of Montpelier, and Dr. Lowenhayn of Moscow, during their Residence in this Country. To which are added, Observations on the Disease in Edinburgh, and the neighbouring Districts, with numerous Cases and Dissections. By JOHN LIZARS, &c. 8vo, stitched, pp. 72. Edinburgh, 1832.

☞ *This will be noticed in the Periscope of the present number.*

12. Observations in Surgery and Pathology; illustrated by Cases, and by the Treatment of some of the most important Surgical Affections. By WILLIAM JAMES CLEMENT, Surgeon. 8vo, pp. 230. London, 1832.

---

13. A Report of the Royal Dispensary for Diseases of the Ear, Dean Street, Soho Square, from 1817 to 1830. 8vo, stitched, pp. 44. London, 1832.

---

14. On the Enlisting, the Discharging, and the Pensioning of Soldiers. With the Official Documents on the Branches of Military Duty. By HENRY MARSHALL, Deputy Inspector General of Army Hospitals. 8vo, pp. 243. London, 1832.

---

15. A Practical Treatise on the Forms, Causes, Sensibility, and Treatment of Pulmonary Consumption. By EDWARD BLACKMORE, M.D. Physician to the Plymouth Public Dispensary. 8vo, pp. 242. London, 1832.

---

16. Practical Observations upon the Epidemic Cholera at Cork. By D. B. BULLEN, M.D. one of the Physicians to the North Cholera Hospital, &c. 8vo, stitched, pp. 28. London, 1832.

☞ *A sensible pamphlet. We have made an extract from it in another place.*

---

17. Surgical Pathology. A Thesis. By JULES CLOQUET. Translated from the French, by J. W. GARLICK, and W. C. COPPERTHWAITE, M.R.C.S. &c. With Plates. Highley, London, 1832.

☞ *We shall take some notice of this useful little work.*

---

18. Medicina Simplex, or the Pilgrim's Way-book; being an Enquiry into the Moral and Physical Conditions of a Healthy Life and Happy Old Age. With Household Prescriptions. By a Physician. 12mo, pp. 254. London, 1832.

☞ *This little work displays good feeling, good sense, and a well-stored mind.*

---

19. On Health, and the Means of preserving it. A Treatise, in Two Parts, Philosophical and Popular. With an Appendix on Cholera. 18mo, pp. 120. Dundee, 1832.

☞ *A very sensible production by one who, we have reason to know, is the possessor of no inconsiderable talents.*

20. Harveian Oration for 1832; being the Discourse read before the Harveian Society of Edinburgh, on the Fiftieth Anniversary of its Institution. By Rich. Huil, M.D. Fellow of the Royal College of Surgeons, &c. 8vo, stitched. Edinburgh, 1832.

21. Part VII. of the Principles and Practice of Obstetric Medicine, &c. By David D. Davis, M.D. &c. Price 2s. Taylor. London, 1832.

22. A Manual of Percussion and Auscultation. Composed from the French of Meriadic Laennec. By James Birch Sharp, M.R.C.S. Duodecimo, pp. 120. Highley. With a Plate. 1832.

☞ *We can strongly recommend this as one of the best and most portable Vade Mecums of Auscultation that has yet appeared.*

23. Outlines of Medical Botany; comprising Vegetable Anatomy and Physiology, the Characters and Properties of the Natural Orders of Plants, an Explanation of the Linnæan System of Classification, and Tables of Medicinal Plants, arranged in their Linnæan and Natural Orders. By Hugo Reid, Member of the Royal Physical Society and of the Society of Arts, Extraordinary Member, and lately President of the Hunterian Medical Society of Edinburgh. Duodecimo, pp. 390. Edinburgh, 1832.

24. An Essay on the Epidemic Cholera; being an Enquiry into its New, or Contagious Characters; including Remarks on the Treatment; as likewise Tables of the average State of Disease and Mortality, recently occurring in London. By John Webster, M.D. Member of the Royal College of Physicians, London; Physician to St. George's and St. James's Dispensary, &c. 12o, pp. 220. Lon. 1832.

25. The Anatomy of the Brain, with some Observations on its Functions. By Alexander Monro, M.D. To which is prefixed, an Account of Experiments on the Weight and Relative Proportions of the Brain, Cerebellum, and Tuber Annularæ, in Man and Animals, under the various circumstances of Age, Sex, Country, &c. By Sir William Hamilton, Bt.

☞ *Dr. Monro's Anatomy of the Brain, like all his other compositions, is distinguished by the fairest characteristics of disinterestedness and didactic excellency: hereafter, we shall enable our readers to estimate the debt of gratitude they owe to the illustrious Professor, for this additional contribution to the art of anatomy; and especially for his account of the "new and valuable discoveries" of his "much esteemed colleague," whose previous discoveries we may, at the same time, illustrate with analogical excerpts from "experiments on the cauda radicula, and tuber annularæ, in the turnip and potatoes, under the various circumstances of age, sex, country, &c. by Sir Louty Bumpkin, knight."*

26. An Exposure of the Unphilosophical and Unchristian Expedients adopted by Anti-phrenologists for the purpose of obstructing the moral and philanthropical Tendencies of Phrenology; being a Review of "Anti-phrenology; or, Observations to prove the fallacy of a modern Doctrine of the Human Mind. By John Wayte, M. D. "He that disapproves of plain dealing with defamers, might as justly deprecate ungentleness in strangling a ruffian."—*Censorinus.* 8vo, pp. 198. London, and Edinburgh, 1831.

☞ *Dr. Wayte's "Observations" are here examined page by page, paragraph by paragraph, sentence by sentence, and refuted; their unfairness and fallacy are condemned by multiplied evidences; and their spirit is rebuked with the severity due to extraordinary misrepresentation.*

27. Annals of some remarkable Aerial and Alpine Voyages, including those of the Author, &c. By T. Forster, M.B. F.L.S. &c. Octavo, pp. 118, sewed, 1832.

☞ *This pamphlet of Dr. Forster's contains much curious and interesting information, which we hope to notice in our next Number.*

### NOTICE TO CORRESPONDENTS.

The letter respecting an unfair review of Mr. Ingleby's work on Uterine Hæmorrhage, from an old correspondent in the West, has been received, but we cannot notice the criticisms of other journalists. The best antidote to the poison will be found in a full and fair analysis of Mr. Ingleby's valuable work, which will be concluded in our next number.

